# Adhesive small bowel obstruction with familial Mediterranean fever, case series and literature review

**DOI:** 10.1016/j.ijscr.2025.111823

**Published:** 2025-08-19

**Authors:** Naser El-Mefleh, Aya Nakwan

**Affiliations:** a Department of Pediatric Surgery, National Hospital, Aleppo, Syria; b Department of Pediatric Surgery, Deva Hospital, Aleppo, Syria; c Faculty of Medicine, Aleppo University, Aleppo. Syria

**Keywords:** Bowel obstruction, Familial Mediterranean fever, Pediatrics, Adhesive peritonitis, Small bowel obstruction, case series

## Abstract

**Introduction and importance:**

Familial Mediterranean Fever (FMF) manifests in 90 % of patients as recurrent episodes of peritoneal inflammation, mimicking an acute surgical abdomen. These attacks typically resolve spontaneously within 72 h. However, recurrent peritonitis can lead to primary intraperitoneal adhesions even in the absence of prior surgery—a rare but serious complication of FMF that may result in bowel strangulation or volvulus. Early recognition is crucial to prevent life-threatening complications.

**Case presentation:**

We present three pediatric cases of FMF complicated by primary adhesive small bowel obstruction (PASBO). Two patients had a prior FMF diagnosis, while the third was diagnosed postoperatively based on surgical findings.

**Clinical discussion:**

All three patients required surgical intervention for PASBO, with varying degrees of adhesion severity. Early recognition of PASBO in FMF patients is essential to prevent complications such as bowel necrosis.

**Conclusion:**

PASBO should be considered in FMF patients with persistent obstructive symptoms or atypical abdominal pain. Conversely, PASBO in patients with a history of recurrent abdominal pain should raise suspicion for undiagnosed FMF. Increased awareness among pediatric surgeons, gastroenterologists, and pediatricians is critical for timely intervention.

## Highlights

• PASBO is a rare but serious complication of FMF, occurring in approximately 3 % of cases.
• Recurrent peritonitis leads to primary adhesions, even without prior surgery.
• FMF patients with atypical or prolonged abdominal pain should be evaluated for PASBO.
• PASBO in children without prior surgery should prompt consideration of FMF.
• Early surgical intervention may prevent bowel necrosis.

## 1 Introduction

Familial Mediterranean Fever (FMF) is an autosomal recessive auto-inflammatory disorder prevalent among Turkish, Arab, Armenian, and Jewish populations [1]. In 90 % of cases, the first attack occurs before age 20 [2]. The hallmark presentation includes recurrent fever and serositis, with peritoneal inflammation being the most common manifestation (90 % of patients) [3]. While typical FMF attacks resolve within 72 h with anti-inflammatory treatment, recurrent peritoneal inflammation can lead to primary adhesions without prior surgery [4]. This a rare but serious complication that may cause small bowel obstruction (SBO) and even bowel necrosis [5]. Although genetic testing for MEFV mutations is preferred when making an FMF diagnosis, the diagnosis remains clinical, because mutations have varying penetrance and homozygosity cannot always be demonstrated [6]. FMF diagnosis can be made using the Tel-Hashomer clinical criteria, as clarifying in the Table 1 [7]. This case series highlights three pediatric FMF patients who developed PASBO, emphasizing the need for early diagnosis and surgical intervention when conservative management fails. This study complies with the PROCESS 2025 guidelines [8].

**Table 1:** FMF diagnosis criteria [7].

Major criteria	Minor criteria
Typical attacks of (1–4)	1. Incomplete attacks involving the **chest**, **joints**, **or both**
1. Peritonitis (generalized)	2. Leg pain with exertion
2. Pleuritis (unilateral) or pericarditis	3. Favorable response to colchicine
3. Monoarthritis (hip, knee, or ankle)	
4. Fever only	
Incomplete attacks of the abdomen	
*Diagnosis requires ≥1 major criterion *or* ≥2 minor criteria. ➢ **Typical attacks** are recurrent (at least 3 episodes of the same type), febrile (rectal temperature ≥38 °C), and short in duration (12 to 72 h). ➢ **Incomplete attacks** are painful and recurrent. They differ from typical attacks in 1 or 2 features: 1. Temperature is normal or <38 °C. 2. Attack lasts longer or shorter than a typical attack but is not less than 6 h long and lasts no more than 1 week. 3. No signs of peritonitis during abdominal attacks. 4. Abdominal attacks are localized. 5. Arthritis occurs in a joint other than the hip, knee, or ankle.	

## 2 Methods and results

Cases were reviewed with attention to diagnostic criteria, imaging, and surgical outcomes.

### 2.1 Case 1

**Fig. 1:**
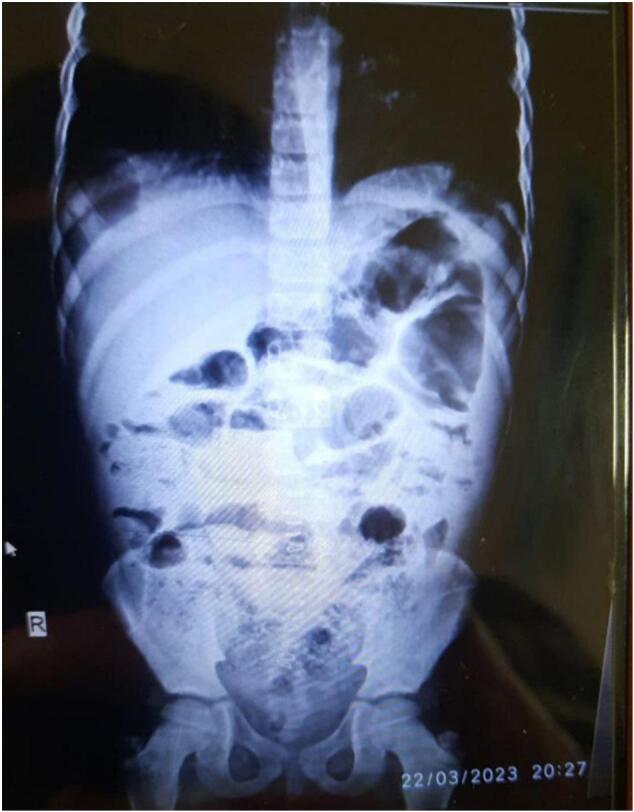
Abdominal X-ray showing multiple dilated small bowel loops with air-fluid levels, consistent with small bowel obstruction.

A 10-year-old boy with a confirmed diagnosis of FMF (diagnosed by a pediatrician using the Tel-Hashomer criteria and a favorable response to colchicine) presented with acute abdominal pain and imaging findings (Fig. 1) suggestive of bowel obstruction (initially misdiagnosed as appendicitis on CT). Initial laparoscopy revealed omental appendiceal adhesions, but his symptoms persisted after an appendectomy. *Re*-exploration identified diffuse adhesions, most severe around the ileum and splenic flexure. Adhesiolysis was performed, and he remained asymptomatic at the 15-month follow-up.

### 2.2 Case 2

**Fig. 2:**
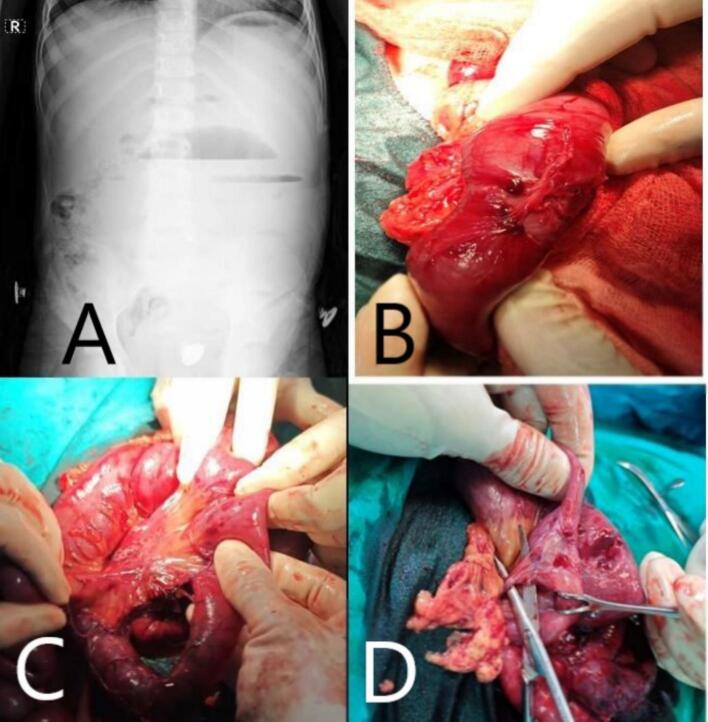
Radiological and intraoperative findings. (A): Plain abdominal X-ray showing multiple air-fluid levels, dilated bowel loops and an absence of distal gas suggestive of small bowel obstruction. (B): Intraoperative view demonstrating dilated, proximal small bowel with an abrupt transition point resulting by adhesions. (C): Intraoperative view demonstrating distended proximal segment adjacent to a collapsed distal bowel; and mesenteric injury. (D): Intraoperative view demonstrating bands narrowing the bowel.

A 6-year-old girl presented with acute bowel obstruction and a two-year history of recurrent abdominal pain and fever associated with elevated CRP and leukocytosis without a prior definitive diagnosis. Imaging (Fig. 2A) finding revealed SBO. During laparotomy, the appendix appeared normal, but upon investigating a Meckel's diverticulum, severe adhesions (Fig. 2B) were encountered, necessitating wound extension. Due to extensive adhesions throughout the small intestine, the mesentery of an ileal loop was injured (Fig. 2C). The decision was made not to resect the loop, and she was discharged on day five. However, vomiting recurred after two weeks, accompanied by poor weight gain.

A second laparotomy revealed a strictured segment at the previously injured mesenteric site, along with diffuse adhesions (Fig. 2D), particularly near the ligament of Treitz. Adhesiolysis was performed, and the narrowed segment was resected with anastomosis. She was discharged after one week and remained asymptomatic at the 6-month follow-up, with normal weight gain. Based on her clinical history (Tel-Hashomer criteria), a diagnosis of FMF was confirmed.

### 2.3 Case 3

**Fig. 3:**
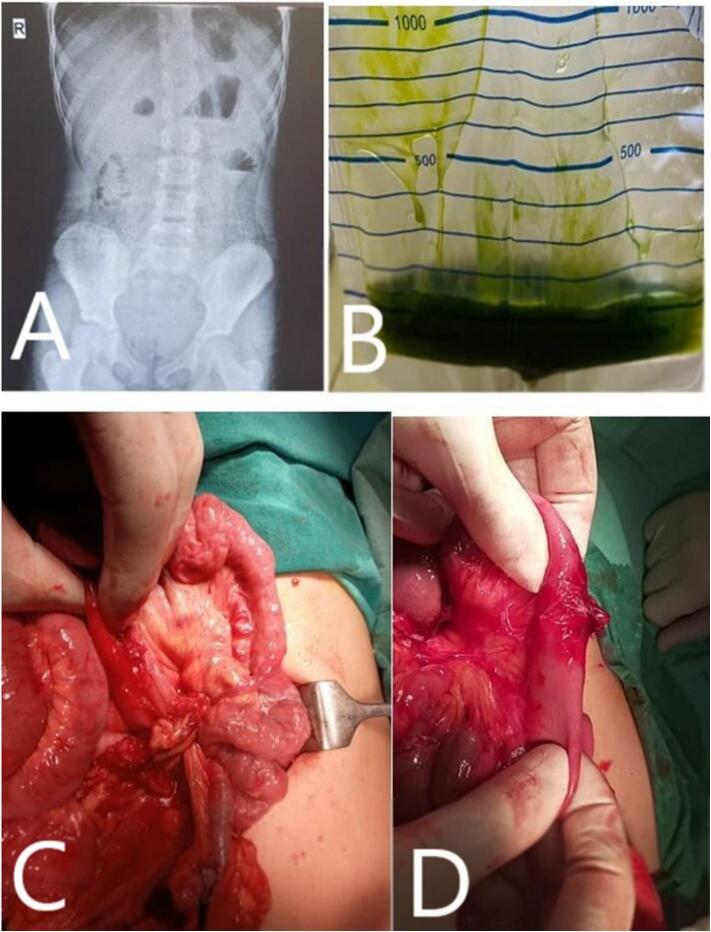
Radiological, clinical, and intraoperative findings. (A) Plain abdominal X-ray showing multiple air-fluid levels suggestive of small bowel obstruction. (B) Greenish bilious aspirate obtained via nasogastric decompression. (C and D) Intraoperative images showing dense inflammatory adhesions between small bowel loops without prior surgical history, consistent with a primary adhesive process.

A 7-year-old girl (previously diagnosed with FMF by a pediatrician, with a positive family history of FMF and a consistent response to colchicine) presented with proximal SBO (Fig. 3A), manifesting as bilious emesis (Fig. 3B) and epigastric pain. The ultrasound showed no mass and presence of dilated proximal intestinal loops at the expense of the stomach and the beginning of the intestine, consistent with the findings of the plain radiograph and gasless abdomen. Laparotomy revealed adhesive bands (Fig. 3C and D) near the ligament of Treitz (proximal jejunum), which were lysed. She recovered without complications and remained asymptomatic at 6-month follow-up.

In our series, surgical intervention was strictly reserved for cases with unresponsive to conservative management and imaging findings or clinical evidences.

## 3 Discussion

Both familial Mediterranean fever (FMF) and peritoneal adhesion-related small bowel obstruction (PASBO) can mimic acute abdominal conditions. However, the relationship between PASBO and FMF remains neither well-defined nor thoroughly evaluated in the literature. The reported incidence of PASBO in FMF patients is approximately 3 %, with no associated mortality [9]. Nevertheless, this life-threatening surgical emergency should be considered in the differential diagnosis of acute abdominal attacks in FMF patients [5]. FMF is an autosomal recessive auto-inflammatory disorder characterized by serositis affecting the peritoneum, pleura, and synovial bursa of joints. While rare globally, it is highly prevalent among individuals of Jewish, Armenian, Arab, and Turkish descent. In the Mediterranean region, the incidence reaches 1 in 200 in the general population [9]. Approximately 80–90 % of FMF attacks occur before the age of 20, with intervals between episodes ranging from days to years [10]. The disease results from mutations in the MEFV gene, located on chromosome 16p13.3, which comprises 10 exons. This gene encodes a 781-amino acid protein (pyrin or marenostrin) that modulates the transcription of intranuclear peptides involved in inflammation [11,12]. Notably, FMF patients undergo abdominal surgeries at a significantly higher rate than healthy controls (29 % vs. 8.7 %), with appendectomy being the most common procedure (up to 26 % of cases), often performed before an FMF diagnosis is established [13]. Another study reported that appendectomy accounts for 19 % of surgical interventions in FMF patients [14]. Differentiating between an FMF-related abdominal attack and acute appendicitis can be challenging. Procalcitonin levels (cut-off: 0.5 ng/mL) have demonstrated 89 % sensitivity and 62 % specificity in distinguishing these conditions, with elevated levels typically observed in appendicitis [15].

As previously noted, FMF patients undergoing surgery are at increased risk of adhesion formation and subsequent SBO [16]. More than 95 % of FMF patients experience peritoneal involvement, which often mimics an acute abdomen but typically resolves spontaneously within 72 h, and recurrent peritonitis may lead to primary intraperitoneal adhesions, even in the absence of prior abdominal surgery [17]. Therefore, if an atypical pain episode fails to resolve spontaneously, surgeons must consider PASBO due to the associated risk of strangulation and bowel necrosis [18,19]. Colchicine remains the cornerstone of FMF treatment, preventing recurrent attacks and amyloidosis in most cases. For colchicine-resistant patients, IL-1 inhibitors are the preferred alternative [20,21]. However, adhesion formation may still occur despite colchicine therapy [22]. FMF patients are at higher risk than the general population for developing BO, either spontaneously or as a postoperative complication. Clinicians should maintain a high index of suspicion for this complication when evaluating FMF patients in the emergency setting [22]. Distinguishing between a typical FMF attack and PASBO is challenging, and delayed diagnosis can lead to severe consequences. Early abdominal CT imaging should be considered if symptoms do not improve promptly, as this may facilitate earlier PASBO diagnosis and surgical intervention, thereby preventing intestinal necrosis [23,24]. Current English-language literature on PASBO in FMF remains limited, underscoring the need for greater clinical awareness. Therefore, FMF patients presenting with abdominal pain and ileus require meticulous evaluation for possible PASBO. Additionally, patients with PASBO but no prior abdominal surgery should be assessed for underlying auto-inflammatory disorders, particularly if they exhibit recurrent abdominal pain with fever.

We found 10 cases in literature review (1958–2025), highlighting that PASBO in FMF is increasingly recognized even in non-endemic regions due to migration [5,17,19,22,[25], [26], [27], [28], [29], [30], [31]]. Eight of them during the last 18 years, 5 articles from areas where FMF is common and 3 ones where it is not common, as in France, Germany, and Kuwait, and this demonstrates the increasing importance and frequency of this association and its early diagnosis throughout the world, not just the areas where it is common. Due to travel, tourism, and immigration movements, some cases were diagnosed in areas with a low FMF prevalence. The small sample size, retrospective design and single-center data limit generalizability. Our findings highlight that Familial Mediterranean Fever (FMF) must be considered a key differential diagnosis in cases of acute abdomen, irrespective of geographic prevalence. Importantly, while surgical intervention can be lifesaving for FMF-related peritonitis or bowel obstruction, clinicians must remain cognizant of its potential complications—particularly adhesion formation and disease exacerbation. Postoperative adhesions may necessitate repeated surgical interventions, thereby perpetuating a detrimental cycle of morbidity (Table 2).

**Table 2:** literature review of documented cases of PASBO associated with FMF.

The year	Details
1966	2 FMF cases who died of PASBO and strangulation [31].
1980	3 FMF cases with PASBO [17].
1995	A review of 355 FMF and PASBO incidences found 3 % [5].
2006	2 FMF cases with PASBO and strangulation [19].
2007	8 of 471 FMF (2 %) with PASBO [22].
2011	A 16-year-old male with PASBO and strangulation [26].
2014	A 7-year-old female with PASBO and volvulus [27].
2018	6-year-old male with PABSO [28].
2019	17-year-old female with proximal PABSO [29].
2021	17-year-old female with proximal PABSO [30].
2023	FM case with PASBO [25].

## 4 Conclusion

PASBO is a rare but critical complication of FMF that requires high clinical suspicion. Early diagnosis and timely surgical intervention can prevent catastrophic outcomes. Larger studies are needed to define PASBO incidence and preventive strategies.

## Abbreviations

PASBO: primary adhesive small bowel obstruction
FMF: familial Mediterranean fever
SBO: small bowel obstruction

## Author contribution

Study conception and design: Naser El-mefleh

Data acquisition: Naser El-mefleh

Analysis and data interpretation: Naser El-mefleh, Aya Nakwan

Drafting of the manuscript: Naser El-mefleh, Aya Nakwan

Critical revision: Naser El-mefleh

## Consent

Written informed consent was obtained from the patients' parents/legal guardian for publication and any accompanying images. A copy of the written consent is available for review by the Editor-in-Chief of this journal on request.

## Ethical approval

Approved by institutional review board.

## Guarantor

Naser el-mefleh.

## Research registration number

Not applicable.

## Funding

None.

## Conflict of interest statement

None.

## Data Availability

Available upon request.
